# Infancy, childhood, and puberty on the Silk Road revealed with isotopic analysis of incremental dentine

**DOI:** 10.1038/s41598-022-24119-3

**Published:** 2022-11-14

**Authors:** Tingting Wang, Dong Wei, Bing Yi, Hongen Jiang, Wenying Li, Yaowu Hu, Benjamin T. Fuller

**Affiliations:** 1 Department of Anthropology, School of Sociology and Anthropology, Sun Yat-Sen University, Guangzhou, 510275 People’s Republic of China; 2 Laboratory of Human Evolution and Archaeometry, Sun Yat-Sen University, Guangzhou, 510275 People’s Republic of China; 3 College of Archaeology, Jilin University, Changchun, 130012 People’s Republic of China; 4 School of Humanities, Xiamen University, Xiamen, 361005 People’s Republic of China; 5 Department of Anthropology and Archaeology, University of Chinese Academy of Sciences, Beijing, 100049 People’s Republic of China; 6 Institute of Archaeology and Cultural Relics of Xinjiang Uyghur Autonomous Region, Urumqi, 830000 People’s Republic of China; 7 Institute of Archaeological Science, Fudan University, Shanghai, 200433 People’s Republic of China; 8 Department of Cultural Heritage and Museology, Fudan University, Shanghai, 200433 People’s Republic of China; 9 Géosciences Environnement Toulouse, UMR 5563, CNRS, Observatoire Midi-Pyrénées, Toulouse, France

**Keywords:** Biochemistry, Biogeochemistry, Environmental social sciences

## Abstract

Childhood is a unique phase in human life history, in which newborns are breastfed and weaned, and are progressively familiarized to adult diets. By investigating dietary changes from infancy to adolescence, valuable information regarding past cultural behaviors and aspects of human lives can be explored and elucidated. Here, in conjunction with published isotopic results of serial dentine (n = 21) from Yingpan Man, new δ^13^C and δ^15^N results are obtained from 172 samples of incremental dentine from 8 teeth of 8 individuals of the Yingpan cemetery, located in Xinjiang, China. The δ^13^C values range from – 18.2 to – 14.6‰ with a mean ± SD value of – 16.3 ± 0.9‰, and the δ^15^N results range between 13.4 and 19.9‰ with a mean ± SD value of 16.0 ± 1.4‰. This indicates that the childhood diets were mixtures of C_3_ and C_4_ dietary resources and were clearly influenced by breastfeeding and weaning practices. In particular, the findings indicate that there were significant inter-individual differences in terms of the timing and duration of breastfeeding and weaning practices as well as childhood dietary practices at Yingpan. For instance, three individuals were exclusively breastfed after birth, while, two individuals and Yingpan Man were not. In addition, the post-weaning diets of most Yingpan individuals were relatively stable, but one individual and Yingpan Man displayed clear evidence of increased consumption of C_4_ foods, likely millet, during late and post-weaning periods. Further, 7 individuals had unique dietary changes between 9 to 14 years old. Potential factors related to this are presented from the perspective of changes in social roles that might be caused by their early participation in the social division of labor or puberty and marriage.

## Introduction

Breastfeeding refers to the process by which infants consume breastmilk, a primary nutritional source that contains not only essential nutrients such as fat, protein, carbohydrates and variable minerals and vitamins, but also substances that help protect newborns from infection and inflammation, as well as to help them develop a healthy immune system and gut microbiomes^1^. This process is proven to be very beneficial to the child and the mother in terms of both short-term and long-term health, and also for the two to bond. Thus, today, the World Health Organization (WHO) recommends that infants begin breastfeeding within the first hour of life, be exclusively breastfed for the first six months, and continue breastfeeding until at least 2 years old^2^. However, at the age of 6 months old, breastmilk alone is no longer sufficient enough to provide the required amounts of nutrients for a child. Appropriate complementary foods are suggested to be introduced to the child to maintain the expected growth rate, remain healthy and well nourished, whilst the supply of mother`s breastmilk is gradually withdrawn and diminished, and this process is known as weaning^2^. Thus, breastfeeding, weaning and childhood dietary practices are key episodes in an individual`s life course^3^. They play an important role in the healthy growth and development of a child, and are crucial for the continuity of a family, culture and even the survival of the human species^4,5^. However, the processes of breastfeeding, weaning and childhood food ways are conditioned by not only the basic physiological needs of the child, but also multiple social, economic and ecological factors beyond the child itself, such as parental education, work requirements, religious beliefs, subsistence strategies, mobility behaviors, availability of suitable infant foods, living environments, social ideas, identities and even status^3,4,6^. In this context, childhood dietary practices reflect the deep entanglement of biology and culture, and bonds together the material and in-material aspects of human lives^5,7^. Thus, there is a high degree of variability in terms of the timing and duration of breastfeeding and weaning practices, as well as the composition of childhood food sources between different human groups and societies^3^.

The dynamic nature of childhood dietary practices highlights the pluralistic way in which “dietary”, “biological” and “social” aspects of the body are viewed, and calls for the incorporation of childhood social theory, especially social age categories, into pre-adulthood studies^7,8^. According to historical literature, the earliest human life phase in ancient China was known as “*Qiang Bao*” (baby in swaddling clothes) which represents infancy or from birth to ~ 1 year of age^9^. The second phase known as “*Hai Ti*” (child that needs to be carried) is early childhood and it spans from ~ 2 to ~ 3 years old^10^. The third phase of middle childhood is named “*Chui Tiao*” (meaning putting hair down) and it has different age ranges for males (~ 3 to ~ 9 years old) and females (~ 3 to ~ 8 years old)^11^. The fourth life stage known as “*Zong Jiao*” (hair in two knots or buns) is the late childhood period and it spans the age range of ~ 9 to ~ 14 years old for males and ~ 8 to ~ 14 years old for females^12^ (Table S1). Subsequent to which, females were considered marriageable with the ceremony of “Ji Ji” (hair tied up with hairpin) at 15 years old^13^, and males were considered adults with the ceremony of “Guan” (hair tied up with tuinga) at 20 years old^13,14^. It is notable, the transition between different life stages is signified by a change of hair style and outlook in ancient China^13,14^, which suggests that the change of an individual`s life course is accompanied with a variation in the way they were viewed by the other members of the community, and this could have led to changes in their social roles and lifeways. Thus, by incorporating these social age categories into the investigation of potential dietary changes throughout infancy and childhood, valuable information regarding past cultural behaviors and aspects of human lives can be explored and elucidated for ancient China^3,4,6^.

## Reconstructing human early life histories with isotopic analysis of serial dentine

Previous research about past breastfeeding, weaning and childhood dietary practices were mainly based on limited evidence inferred from historical documents and archaeological findings^5,7,15^. However, in past decades, isotopic research has emerged as an effective means for this area of research. Based on studies of nail clippings and hair segments of modern human mother and infant pairs, exclusively breastfed infants are elevated in δ^13^C (~ 1‰) and δ^15^N (~ 2–3‰) values compared to their mothers, and this is caused by the fact that infants are essentially consuming their mother`s tissues through breastmilk ingestion^16,17^. However, this ^13^C-enrichment quickly declines with the consumption of supplementary foods while the ^15^N-enrichment declines over the course of the weaning process^17^. Thus, by comparing the isotopic values of bones that are formed during childhood (e.g. bones from known age nonadults) and adulthood it is possible to investigate past breastfeeding and weaning practices^5,18–25^. However, this approach can be problematic due to the difficulties in estimating osteological age in nonadults, the lack of discrete temporal resolutions in bone turnover rates as well as the osteological paradox regarding the representativeness of non-adults` bones, or in other words, “non-survivals”, in mortuary contexts^4,6,7,15^.

**Figure 1:**
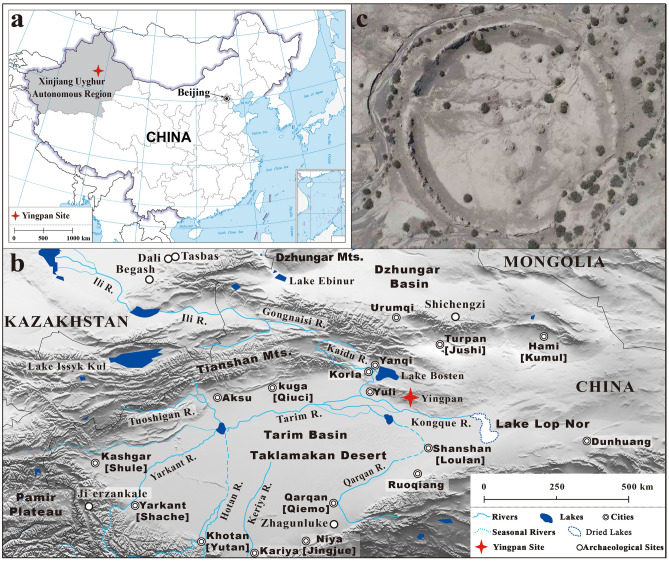
Map showing the location of Yingpan. (**a**) Location of Yingpan in Xinjiang, China; (**b**) Distribution of archaeological sites and ancient cities in Xinjiang; (**c**) Arial view of Yingpan city. (Maps were generated in Standard Map Service (http://bzdt.ch.mnr.gov.cn) and GMT V.5.2.1. The original pictures of the site was previously published^70^ and provided by Wenying Li. The final layout was created using Adobe Illustrator CC 2019V.23.1.1.).

The isotopic study of teeth overcomes many of these problems as different teeth develop at different ages of life and are retained into adulthood, which enables an investigation concerning the early life histories of the “survived” adults^20,26–29^. In particular, significant methodological improvements have been achieved in recent years with the development of incremental sampling of human dentine^22,29–32^. Dentine grows diachronically from the dentin-enamel junction toward the root and thus records and retains time bound isotopic signal derived from the period of tooth formation^30^. In particular, the resolution of such longitudinal studies has been refined in the past 20 years by increasing the number of intra-tooth samples from ~ 3 to > 20 sections^22,33,34^. Thus, isotopic analysis of serial dentine is now widely accepted as a routine method for the investigation of past breastfeeding, weaning and childhood dietary practices^1,3,4,6,7,22,31,33–40^. Here, building on our previous research of plants, human bone, hair, muscle and scalp samples from the Yingpan cemetery site in Xinjiang, China^41,42^, a detailed investigation of the breastfeeding, weaning and childhood dietary practices of this Silk Road population is carried out with the application of isotopic analysis of serial dentine of human teeth (Fig. 1).

The Yingpan site which is located in the northeastern portion of the Tarim Basin was an important Han to Jin Dynasty (206 BCE – 420 CE) city that was situated on a crossroad of the Silk Road (Fig. 1)^43–45^. The ruins of this city were first visited by western explorers in the late 19^th^ and early twentieth centuries^46^. However, it was not systematically excavated until a century later in the 1990s^46–48^. According to the excavation reports, the site of Yingpan includes not only the ruins of the city, but also the remains of a Buddhist temple, a military beacon tower, a public cemetery, farmlands as well as irrigation ditches^46,48^. So far, more than one hundred burials have been excavated in the cemetery, and thousands of graves goods were recovered^46,48^. These grave goods are remarkably diverse in terms of culture, technology and material, and clearly reflect the interaction between humans from different regions of the Eurasian continent^41^. In this context, the Yingpan cemetery site provides an excellent range of bio-archaeological materials for the investigation of human early life histories along the Silk Road during the Han to Jin Dynasty.

## Results

**Figure 2:**
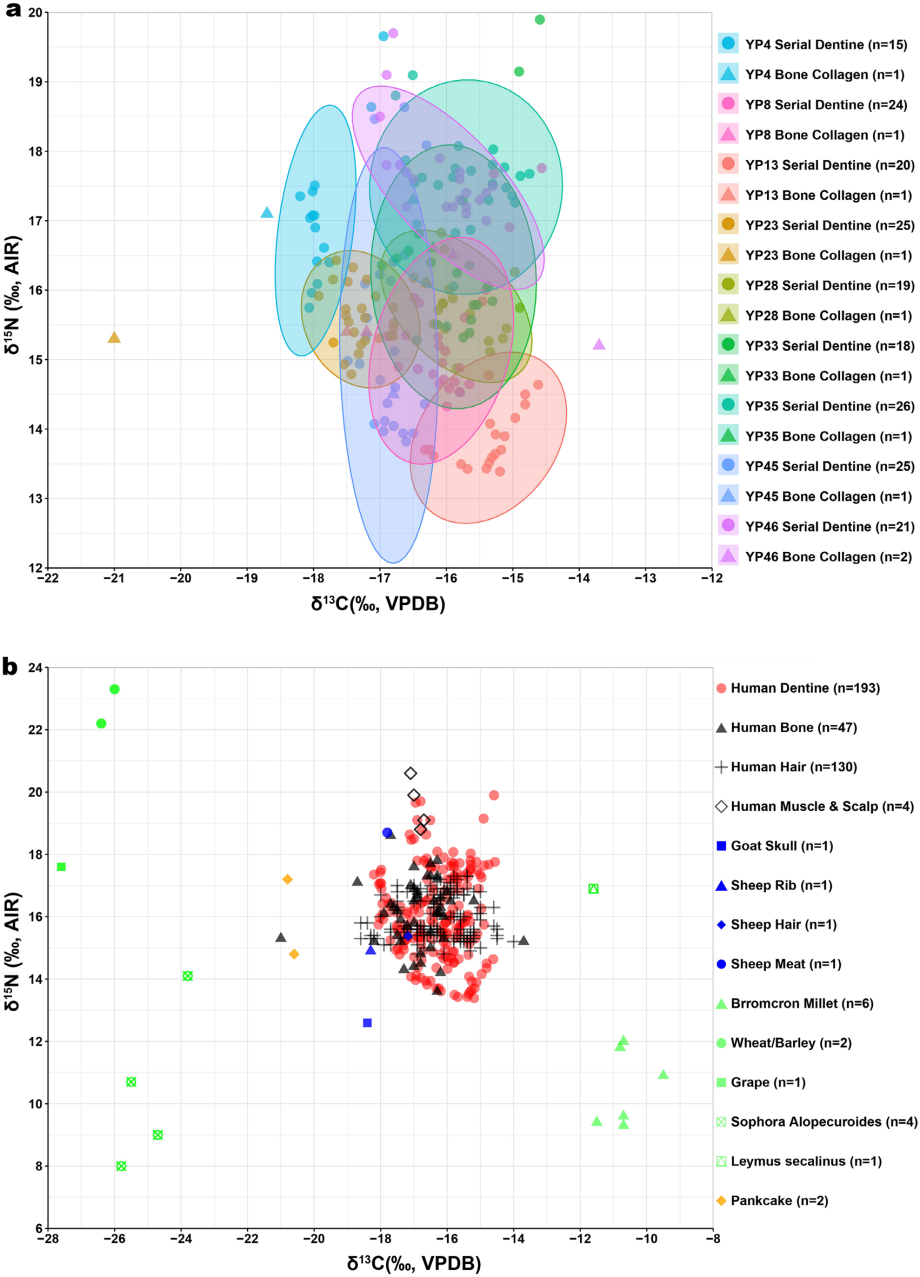
(**a**) Bi-variate scatter with 95% prediction confidence ellipses showing the isotopic results of human serial dentine (this study) and bone collagen from Yingpan^41^; (**b**) Scatter showing the isotopic results of human dentine, bone, hair, muscle, scalp, and associated animal and plant remains from Yingpan^41^. (The plots were generated using RStudio V.1.4.1717).

The isotopic results are shown in Fig. 2a and listed in Table S2. The tooth dentinal sections produced δ^13^C values that range from – 18.2 to – 14.6‰ (mean ± SD value of – 16.3 ± 0.9‰, n = 172) as well as δ^15^N values that range from 13.4 to 19.9‰ (mean ± SD = 15.8 ± 1.4‰, n = 172). This indicates that the childhood diet of these Yingpan individuals were mixtures of C_3_ and C_4_ dietary resources and was influenced by breastfeeding and weaning practices^30^. Diachronically, the δ^13^C values of the incremental dentine showed clear fluctuations between C_3_- and C_4_-based foods during the period of tooth formation (Table S2). While, the δ^15^N results are exceptionally elevated, they are reasonable according to previous research in Xinjiang^49–51^ and the baseline isotopic results of plants and animals from Yingpan (Fig. 2b)^41^.

## Discussion

**Figure 3:**
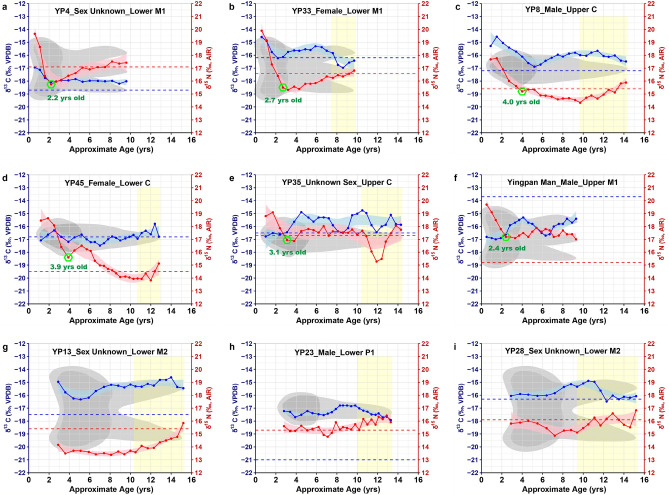
δ^13^C (blue) and δ^15^N (red) results of serial dentine (solid line) and bone collagen (dashed line) from individuals of the Yingpan cemetery^41^. Notes: (1) approximate age of breastfeeding cessation is labelled with green hexagon; (2) sex and tooth element is detailed and specified in the sub-titles; (3) yellow shade highlights the late childhood dietary change observed; (4) regressive curves (blue & pink shades) are estimated with the integrated smoothness model of LOESS (Local Polynomial Regression Fitting) using R. (The original plots were generated using RStudio V.1.4.1717. The final layout was created using Adobe Illustrator CC 2019 V.23.1.1).

Together with the published isotopic results of serial dentine from Yingpan Man (labeled as YP46 or YP Man; n = 21), a total number of 193 samples of dentinal collagen from 9 teeth of 9 individuals are included here for discussion (Figs. 2, 3). These teeth span a broad age range from birth to ~ 15.5 years old, and this corresponds to four different social age periods of an individual`s life as documented in Chinese historical literature^14^ (see Supplementary Table S1). Thus, here, the serial dentine isotopic results are grouped and investigated based on the social age periods of these individuals, and this includes mainly the infancy and early childhood period where breastfeeding and weaning generally occurs (from birth to ~ 3 years old), the middle childhood period (from ~ 3 to ~ 8 years old) upon which post-weaning diet varies, as well as the late childhood period or adolescence (from ~ 8 years old onwards) where significant changes associated with social roles might have occurred.

### Yingpan Silk Road breastfeeding and weaning practices

As displayed in Fig. 3a–f, the isotopic values of serial dentinal sections from the canine (C) and first molar (M1) of the Yingpan humans (YP4, YP33, YP8, YP45, YP35, Yingpan Man) exhibited dramatic changes from birth to ~ 4 years of age. This reflects the duration of breastfeeding and weaning practices for these individuals^17,22^. Archaeological evidence for breastfeeding and weaning practices is rarely preserved in ancient Xinjiang. However, two feeding vessels, one made of goat breast skin and the other made from an ox horn, were previously discovered with a 10-month old infant mummy in Zhagunluke cemetery (located in the southern Tarim Basin, ~ 460 km from the Yingpan cemetery)^43,52^. This infant is named “Qiemo Baby” and dates to ~ 1000 BC. Residues of cereal grains were found in the feeding vessel made from the ox horn and this indicates that cereal foods, such as wheat, barley and millet, were likely used as supplementary weaning foods in ancient Xinjiang in the form of porridge^44,53^. Moreover, historical documents such as the Kharosthī scripts provide additional evidence that children were also fed with animal milk in ancient Xinjiang. To be specific, it is recorded in some of the Kharosthī scripts discovered in Shanshan (a Han Dynasty kingdom located in ancient Xinjiang, ~ 250 km from Yingpan) that a “milk fee” of cattle or camel was paid to the adoptee when children were adopted^54,55^. Thus, there may have been a common tradition of feeding infants with liquid foods such as animal milk or porridge made of cereal grains during the weaning process in ancient Xinjiang. For which, more detailed information is embodied in the dentinal isotopic results of these Yingpan individuals.

As shown in Fig. 3a, the first four 1 mm dentinal sections from the M1 of individual YP4 exhibited a steady decrease in both δ^15^N (~ 4.0‰) and δ^13^C (~ 1.2‰). This indicates that individual YP4 was exclusively breastfed after birth, and that supplementary weaning foods were incorporated into his/her diet since the age of ~ 0.6 years old, and that this individual was fully weaned off breastmilk by the age of ~ 2.2 years old^16,17^. In particular, in consideration of the large range of ^15^N-depletion (~ 4.0‰) during weaning, as well as the same decrease observed for the δ^13^C and δ^15^N, the data presented here suggest that the supplementary weaning foods consumed by individual YP4 likely contained significant amounts of proteins from lower trophic levels^17^. In addition, individual YP33 also displayed similar patterns of exclusive breastfeeding by the age of ~ 0.6 years old as the δ^15^N values of the serial dentine from her M1 steadily decline by ~ 4.4‰ from the 1st to the 6th serial sections whereas the δ^13^C values decrease by ~ 1.6‰ from the 1^st^ to the 4^th^ serial sections (Fig. 3b). This indicates YP33 was fully weaned off breastmilk by the age of ~ 3.2 years old. In particular, the fact that her dentinal δ^13^C results decreased faster than her dentinal δ^15^N results suggest that the supplementary weaning foods consumed by individual YP33 were probably more rich in carbohydrates from lower trophic levels, such as C_3_ cereals like wheat and barley^17^.

Additional evidence for exclusive breastfeeding during early infancy is also seen in individual YP8. Specifically, the dentinal δ^13^C values of the first two 1 mm sections from the upper canine of individual YP8 increase by ~ 0.7‰ while the δ^15^N values increase by ~ 0.1‰ (Fig. 3c). Subsequently, the δ^15^N results of his serial dentine steadily decrease by ~ 2.6‰ from the 2^nd^ to the 6th sections, whereas the δ^13^C values decrease by ~ 2.3‰ from the 2nd to the 8th dentinal sections. This indicates that YP8 was exclusively breastfed after birth, and that he was gradually and slowly weaned off breastmilk from the age of ~ 1.5 to ~ 4.0 years old. In particular, this individual likely consumed significant amounts of C_3_ foods, such as wheat, barley and animal milk, during and soon after weaning.

In contrast, the isotopic profiles of individuals YP45, YP35 and Yingpan Man did not exhibit evidence for exclusive breastfeeding^16,17^. Specifically, the δ^15^N values of the upper canine of individual YP45 firstly increase by ~ 0.1‰ from the 1st to the 2nd dentinal sections and then steadily decrease by ~ 3.0‰ from the 2nd to the 5th serial sections (Fig. 3d). However, the δ^13^C values of the corresponding dentinal sections fluctuate between – 17.1 and – 16.3‰ with a mean ± SD value of – 16.8 ± 0.4‰ (n = 5), which is similar to both the δ^13^C results of the serial dentine formed later (mean ± SD = – 16.8 ± 0.4‰; n = 20) as well as the δ^13^C result of the mandible of this individual (– 16.8‰). Thus, it`s likely that individual YP45 was already introduced to supplementary foods before the age of ~ 1.2 years old, and that she was fully weaned off breastmilk at the age of ~ 3.9 years old^17^. In addition, the δ^15^N values of the first two serial sections from the upper canine of individual YP35 increase by ~ 0.3‰ with a concurrent δ^13^C increase (Fig. 3e). Subsequently, the dentinal δ^15^N values of this individual continuously decrease by ~ 2.2‰ from the 2nd to the 4th serial sections, whereas the δ^13^C results display only minor changes within a range of ~ 0.2‰. This indicates that individual YP35 started to consume supplementary foods since at least ~ 1.0 year of age, and that he/she was fully weaned off breastmilk by the age of ~ 3.1 years old. Similar patterns of steadily declining δ^15^N values coupled with mostly unchanged δ^13^C results are also observed in Yingpan Man. As shown in Fig. 3f, Yingpan Man was not exclusively breastfed after birth and was fully weaned off breastmilk by the age of ~ 2.4 years old as the first five 1 mm serial sections of his dentine continuously decrease by ~ 2.5‰, while the corresponding δ^13^C values vary slightly in a range of 0.4‰^41^.

Overall, though some individuals were exclusively breastfed after birth, and some were not, the dentinal isotopic results of the M1 and C presented here indicates that these individuals were fully weaned off breastmilk somewhere in the age range: ~ 2.2 to ~ 4 years old (mean ± SD = 3.1 ± 0.8; n = 6). This estimated age range for the cessation of breastfeeding at Yingpan is similar to the findings of previous research concerning past weaning practices of ancient China, which includes the Late Neolithic site of Gaoshan where individuals were weaned off breastmilk between the age of ~ 3.5 to ~ 4 years old (mean ± SD = 3.7 ± 0.3; n = 3)^56^, the Western Zhou Dynasty (1122–771 bc) site of Boyangcheng where weaning was completed at around 3 to 4 years old^57^, as well as the Eastern Zhou Dynasty site of Xiyasi (mean ± SD = 3.8 ± 0.7; n = 15) and Changxinyuan (mean ± SD = 3.4 ± 0.9; n = 8) where the individuals were found to stop consuming breastmilk between the ages of ~ 2.5 to ~ 4 years old^5^.

### Yingpan Silk Road post-weaning feeding habits

In contrast to the results from the M1 and C teeth, the samples from the first pre-molar (P1) and second molar (M2) of the Yingpan individuals (YP13, YP23, YP28) failed to capture the detailed information concerning their breastfeeding and weaning practices as these teeth start formation at a later age: ~ 2.5 years old^30^. Thus, as shown in Fig. 3g, h, and i, the δ^13^C and δ^15^N values of the P1 and M2 of these individuals displayed a much smaller range of variability, and this is clearly uncharacteristic of the isotopic signatures of breastfeeding and weaning^16,17^. Instead, it is, to some extent, consistent with the dentinal isotopic patterns of the tested C and M1 teeth that were formed between the age range of breastfeeding cessation and ~ 8 to ~ 10 years old, which together constitute the isotopic profiles for the post-weaning diets of Yingpan.

**Figure 4:**
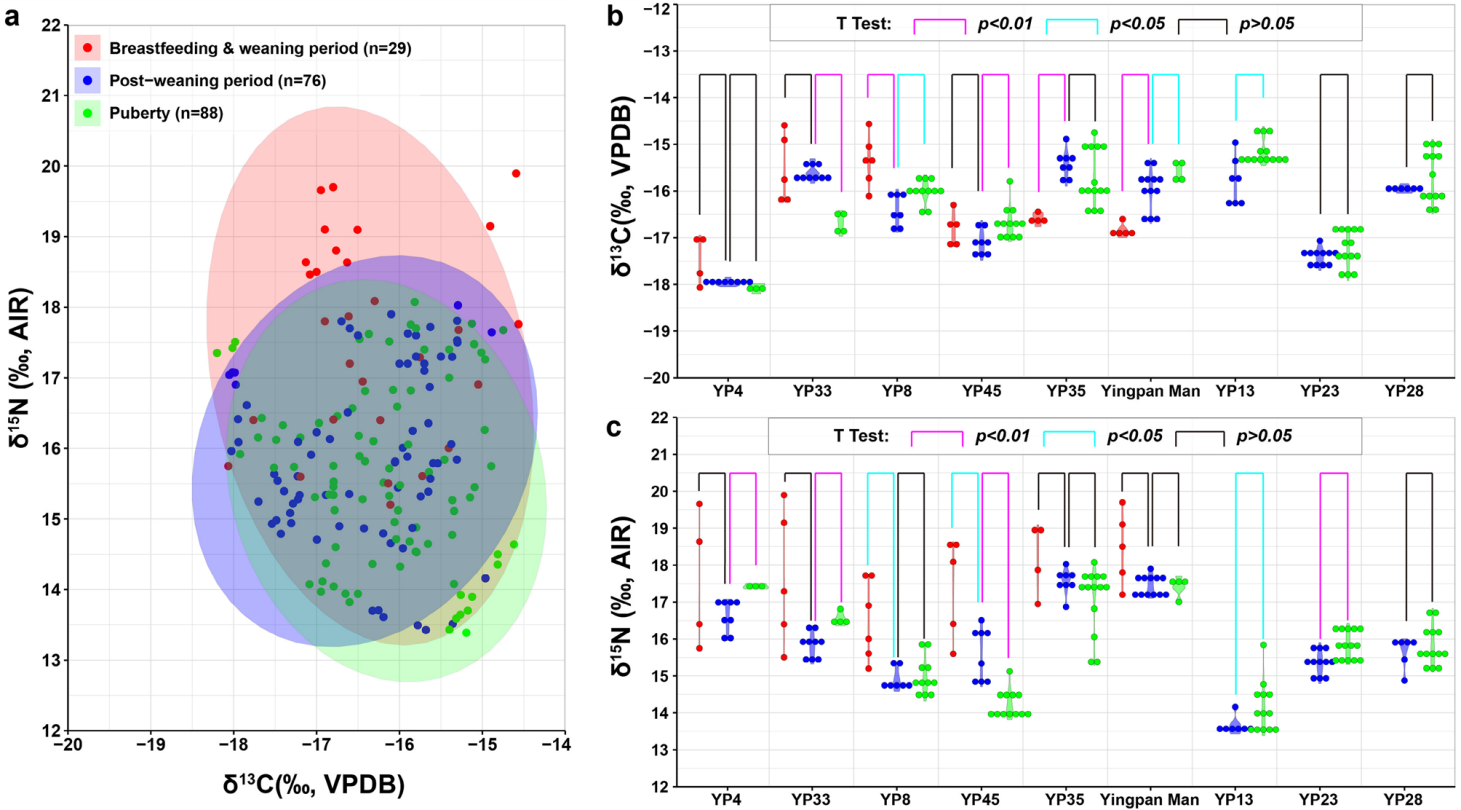
Isotopic comparison between the timeframes of breastfeeding and weaning, the post-weaning and puberty periods. (Results of t test were calculated using R. The original plots of scatter, ellipse and violin were generated using RStudio V.1.4.1717. The final layout was created using Adobe Illustrator CC 2019 V.23.1.1).

As shown in Fig. 4, during the post-weaning periods, the dentinal δ^15^N values of individuals YP35, Yingpan Man, YP13 and YP23 displayed a much smaller range of variability compared to that of the periods before (infancy & early childhood) and afterwards (late childhood). For instance, the δ^15^N values of the lower second molar of individual YP13 were around ~ 13.5‰ for eleven out of the first twelve serial sections studied, or, from ~ 3.6 to ~ 10.5 years old, and the corresponding δ^15^N variation is only ~ 0.4‰ (Fig. 3g). This is evidence of a post-weaning diet that is relatively stable and homogenous in terms of trophic level. In addition, though the dentinal δ^15^N values of individual YP35 firstly increase by ~ 0.7‰ after cessation of breastfeeding, or, from ~ 3.1 to ~ 5.2 years old, the dentinal δ^15^N values of this individual then vary in a small range around ~ 17.5‰ for another twelve serial sections till the age of ~ 10.9 years old (Fig. 3 e). Further, though the dentinal δ^15^N values of Yingpan Man and individual YP23 fluctuated up and down during the post-weaning period, the overall trend of their δ^15^N results were around ~ 17.4‰ and ~ 15.3‰, respectively, from the age of ~ 2.5 to ~ 10 years old (Fig. 3f,h)^41^.

In comparison, the dentinal δ^15^N values of the other Yingpan individuals, including individuals YP4, YP33, YP45, YP8 and YP28, displayed either a larger range of variability or more regular changes that are indicative of a varying diet during the post-weaning periods. As shown in Fig. 3, the dentinal δ^15^N values of individuals YP4 and YP33 progressively increase by ~ 1.8‰ and ~ 1.5‰, respectively, during the post weaning period, or, from ~ 2.5 to ~ 10 years old (Fig. 3a,b). This suggests that these two individuals probably consumed increased amounts of proteins after breastfeeding cessation. In addition, the dentinal δ^15^N values of individual YP45 firstly increase slightly by ~ 0.9‰ in the year after weaning completion (from ~ 3.9 to ~ 5.2 years old), but then steadily decline by ~ 2.6‰ from ~ 5.2 to ~ 11.1 years old. This indicates that the protein consumption of this individual gradually declined during the post-weaning period (Fig. 3 d). Interestingly, individuals YP8 and YP28 also exhibited gradually declining dentinal δ^15^N values during the post-weaning periods, but were clearly in a much smaller range (YP8: ~ 1.1‰; YP28: ~ 1.1‰), and was accompanied with a concurrent δ^13^C increase (YP8: ~ 1.7‰; YP28: ~ 1.2‰). This suggests that individuals YP8 and YP28 probably consumed increased amounts of C_4_ foods from lower trophic levels, such as millets, during this period of life^16,17^.

Coincidently, the overall trend of the dentinal δ^13^C results of most Yingpan individuals, including individuals YP4, YP33, YP45, YP13 and YP23, also exhibited a small range of variability during the post-weaning periods, which indicates that their diets were relatively stable in terms of the consumption of C_3_ or C_4_ foods. However, two exceptional individuals are YP35 and Yingpan Man as their diets appear to have changed frequently and significantly during middle childhood. As shown in Fig. 3e, the dentinal δ^13^C values of individual YP35 steadily increase by ~ 1.7‰ from ~ 2.4 to ~ 4.5 years old, and then stayed at around –15.3‰ for another five dentinal sections till the age of ~ 7.2 years old. This indicates that individual YP35 was switched to a distinct diet as soon as he/she became independent of breastmilk, and this diet clearly contained significant amounts of C_4_ foods, likely millets. Similarly, Yingpan Man also consumed increased amounts of millets during the late and post-weaning period as his dentinal δ^13^C values continuously increase by ~ 1.7‰ from the 3rd to the 9th serial sections, or, from ~ 1.4 to ~ 4.1 years old (Fig. 3f). Notably, YP35 and Yingpan Man also displayed the most frequent δ^13^C fluctuations during middle and late childhood, which together implies that these individuals relied more on millets during the post-weaning periods and also had more significant and frequent dietary changes in the subsequent years^41^.

Here, the finding that millet was increasingly consumed during the late and post-weaning periods is noteworthy as millets were first domesticated in the Yellow River Valleys of China and the tradition of using millets as weaning supplementary foods was originated from the same region^49,56,57^. Evidence in support of this comes from the isotopic research of the Eastern Zhou Dynasty (771–221 BC) site of Xiyasi (n = 15) and Changxinyuan (n = 8) where human serial dentinal collagen produced δ^13^C values (from – 19.2 to –6.7‰) that reflects the consumption of a significant amount of C_4_ foods (likely millets)^5^. In addition, according to Chinese medical sources such as the *Compendium of Materia Medica* (“*Ben Cao Gang Mu*”, compiled by Shizhen Li in 1552 ~ 1578 AD), millets were described to be soft, starchy, nutritious and easy to digest, and were suggested as ideal foods for people who are weak^58^. In this context, millet gruel was utilized as a weaning supplementary food in ancient China, and this tradition still is carried on today in some regions of modern China, e.g. Shanxi and Gansu^56,59,60^.

Further, it is noted that this tradition was also transmitted to other regions of China and Eurasia alongside the Old-World food globalization processes^49,61,62^. For instance, isotopic research on human dentine and bone collagen from the Late Neolithic site of Gaoshan (2500 bc, located in Sichuan Province of China) yielded evidence that C_4_ foods (millets) contributed substantially to human diets during the weaning process and early childhood^36,56^. Similar findings of millet consumption during childhood periods are also detected in samples of human serial dentinal collagen from the Late Antique/Migration Period cemetery of Niedernai (early fifth century AD) in France^34^. These findings hint at the possibility that some Europeans adopted the eastern tradition of using millets as maternal and infant foods via the Silk Road trading networks, and the region of Xinjiang clearly acted as a transitional station for the transmission of such traditions^42^.

### Puberty on the Silk Road at Yingpan

Subsequent to the period of middle childhood, the studied teeth samples of most Yingpan individuals, including individuals YP8, YP33, YP35, YP45, YP13, YP23 and YP28, exhibited dramatical isotopic variations during late childhood, whereas individuals YP4 and Yingpan Man displayed exceptional patterns that are consistent with the isotopic variations of their post-weaning diets. As shown in Fig. 3a, individual YP4 displayed consistent isotopic patterns that reflect increased consumption of proteins during middle and late childhood, and it seems that no significant dietary changes occurred during his/her early life, or, from ~ 2.2 to ~ 9.6 years old. In addition, the dentinal δ^15^N values of Yingpan Man vary slightly during middle and late childhood, while his dentinal δ^13^C values consistently fluctuated between C_3_- and C_4_-based foods, and no significant dietary changes occurred during his late childhood^41^. However, it is notable that the studied teeth of these two individuals are both lower M1, which complete formation at an earlier age (around ~ 10 years old), and this is the reason for their failure to capture the dietary changes during the later periods of late childhood.

In contrast, though the dentinal δ^15^N values of individual YP33 rise continuously from middle to late childhood, the dentinal δ^13^C values of this individual appear to change dramatically from ~ 8 to ~ 10 years old: decrease suddenly by ~ 1.2‰ from ~ 7.6 to ~ 8.6 years old, and then increase by ~ 0.6‰ from ~ 8.6 to ~ 9.7 years old (Fig. 3b). This is clear evidence that individual YP33 was temporarily switched to a diet that contained more C_3_ foods, likely wheat and barley, at around 9 years old. In comparison, individual YP35 exhibited the most significant isotopic changes during late childhood, and these are from the dentinal sections that were formed after 10 years old (Fig. 3 e). Specifically, the δ^13^C values of individual YP35 suddenly decreased from – 14.7‰ (~ 10.5 years old) to –16.4‰ (~ 11.9 years old), and then quickly increased to – 15.1‰ (~ 13.3 years old), whereas the δ^15^N values dramatically decreased from 17.7‰ (~ 10.5 years old) to 15.3‰ (~ 11.9 years old), and then increased to 18.1‰ (~ 13.8 years old) in a short time (Fig. 3e). This is compelling evidence that individual YP35 was temporarily switched to a diet that was ^15^N-depleted and contained more C_3_ foods at the age of ~ 10.5 years old, and this diet lasted till the age of ~ 13.8 years old.

In addition, smaller but prolonged isotopic variations indicative of longer-term dietary changes during late childhood are observed in the dentinal sections of individuals YP8, YP45, YP13, YP23 and YP28. As shown in Fig. 3, the dentinal δ^15^N results of individual YP8 steadily increase by ~ 1.6‰ from ~ 9.7 to ~ 14.2 years old, whereas the corresponding dentinal δ^13^C results decrease by ~ 0.8‰ (Fig. 3c). This concurrent δ^13^C and δ^15^N variation are towards opposite directions, and are in contrast to the isotopic variations observed during the post-weaning period of this individual. This suggests that individual YP8 was introduced to a different diet at the age of ~ 10 years old, and this diet clearly contained more C_3_ foods from higher trophic levels (likely animal protein). Interestingly, similar trends of opposite δ^13^C and δ^15^N variations that are in contrast to the changes of post-weaning diets are also detected in the late childhood of individual YP28 (Fig. 3i). Specifically, the dentinal δ^15^N values of this individual firstly increased by ~ 1.2‰ from ~ 9.4 to ~ 11.1 years old, then fluctuated up and down between 15.5 and 16.6‰ from ~ 11.1 to ~ 15.2 years old. While, the dentinal δ^13^C values firstly decrease by ~ 1.6‰ from ~ 10.5 to ~ 12.3 years old, and then vary slightly in between – 16.5 and – 16.0‰ from ~ 12.3 to ~ 15.2 years old (Fig. 3i). This suggests that individual YP28 was introduced to a diet that is ^13^C-depleted and ^15^N-enriched from ~ 10 years old onwards. Additionally, this dietary pattern is also observed in individual YP23 where increasing δ^15^N alongside decreasing δ^13^C appears in the dentinal sections that were formed after ~ 10 years old (Fig. 3 h). Further, individual YP13 also exhibited steadily increasing dentinal δ^15^N values (~ 2.2‰) from ~ 10.5 to ~ 15.2 years old, but the δ^13^C values of the corresponding dentinal sections changed only slightly around –15.0‰. This indicates that individual YP13 was switched to a diet that contained increased amounts of protein from ~ 10.5 years old onwards (Fig. 3g). Finally, individual YP45 was also switched to a different diet at the age of ~ 11.1 years old as the δ^13^C and δ^15^N values of this individual display concurrent variations in a larger range from this age onwards (Fig. 3d). To sum up, individuals YP8, YP45, YP35, YP13, YP23 and YP28 all displayed unique dietary changes at around 10 to 14 years old, while the dietary patterns of individual YP33 changed significantly at around 9 years old.

Here, the finding that most of the studied Yingpan individuals had significant dietary shifts around the ages of 9 to 14 years old is extraordinary as this was likely associated with dramatical changes of lifeways and food sources that might be caused by the change of an individual`s social role. As discussed earlier and according to Chinese historical literature, individuals between the age of 9 to 14 years old were characterized by having their hair in two knots (“*Zong Jiao*”), a hair style that reduced disturbances in relation to activities or work^12^. In particular, it is interesting that this hair style was also commonly presented in the images of servants and junior workers in ancient China^63^, which implies that it was potentially made for the convenience of work. In addition, recent archaeological research also recovered clear evidence that subadults likely participated in the social division of labor during the Qin and Han Dynasties as their fingerprints and handprints were frequently identified on fictile products, such as the statues of the terracotta warriors discovered in the mausoleum of the First Qin Emperor, and the tomb bricks discovered in Han Dynasty burials of southern China^64^. Thus, it is highly possible that individuals at Yingpan also started to participate in works like trading, traveling, producing, herding or planting, during late childhood, and this likely led to their near universal dietary changes around the age of 9 to 14 years old.

**Figure 5:**
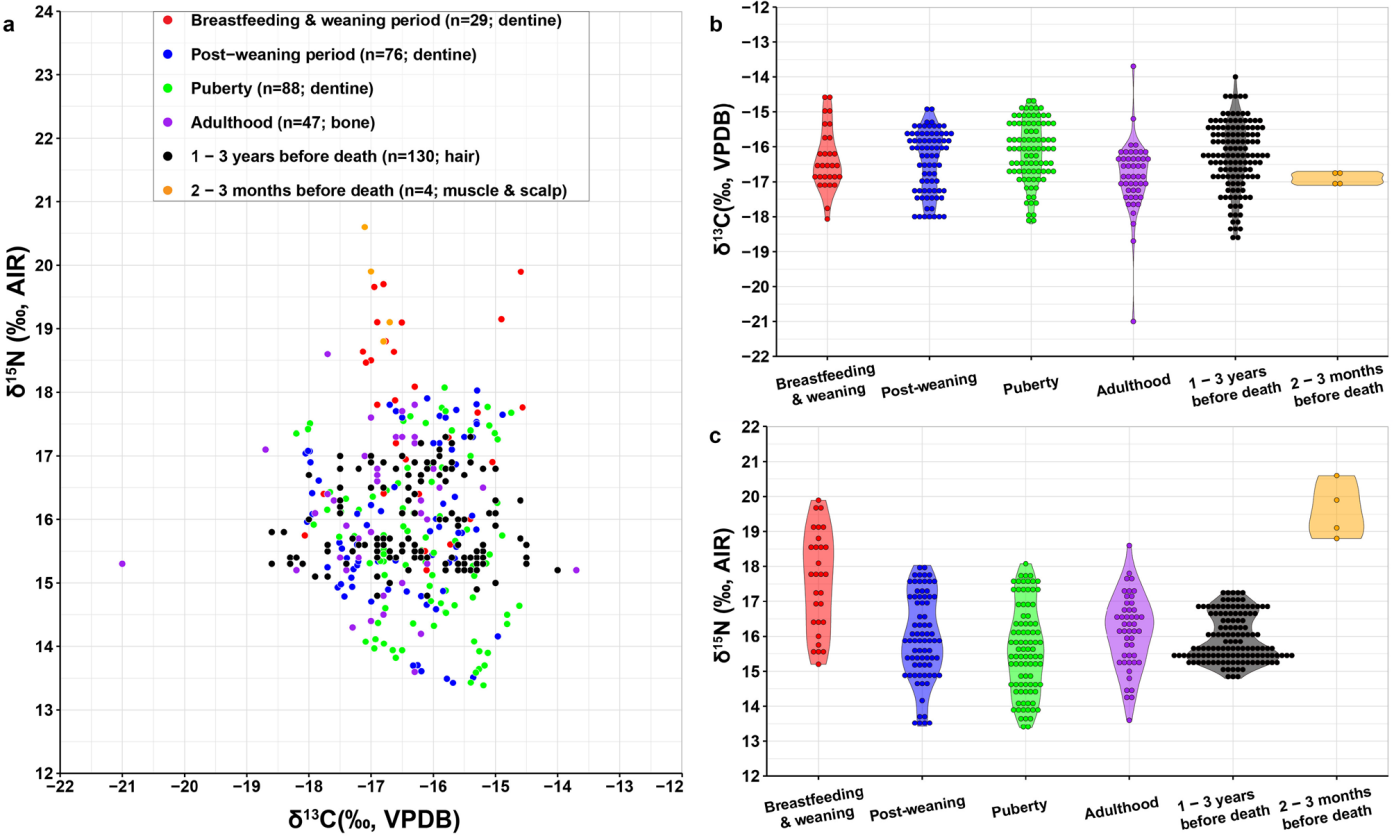
Isotopic comparison between human dentine, bone collagen, hair keratin, muscle and scalp of individuals studied at Yingpan^41^. (The original plots of scatter and violin were generated using RStudio V.1.4.1717. The final layout was created using Adobe Illustrator CC 2019 V.23.1.1).

Notably, as a key spot on the Silk Road, it is predictable that the inhabitants of Yingpan might have traveled to other places for the exchange of goods. Evidence in support of this comes from the grave goods of Yingpan which revealed the global reach of cultures, and the isotopic variability of the individuals^42^. However, it is not likely that these individuals traveled great distances. Specifically, previous research demonstrated that Xinjiang is characterized by a distinctive isotopic niche and the isotopic results of humans from this region are statistically different compared to the humans from other regions. Thus, in combination with previously published isotopic results of human bone, hair, muscle and scalp samples from Yingpan^41^, the serial dentine isotopic results presented suggests that the Yingpan inhabitants were likely local to the Tarim Basin as their δ^15^N values are all exceptionally elevated and their δ^13^C results show a mix of both C_3_ and C_4_ dietary resources (Fig. 5)^49,50^. In summary, these particular Yingpan individuals likely spent most of their lifetimes in and around the Tarim Basin: they were born here, grew up here, and clearly, died here. However, they could have traveled along the Silk Road to certain towns and trading centers in Xinjiang, and this could be a reason for the temporary dietary shifts shown in their serial dentine.

Alternative reasons that might be responsible for the changes of lifeways and food sources at around 9 to 14 years old comes from the perspective of puberty and marriage as this is consistent with the suggested age of marriage during the Han to Jin Dynasties. To be specific, it is recorded in the Chapter of “*Hui Di Ji*” (meaning “biography of Emperor Hui”) in *Han Shu* (meaning “the Book of Han”; written by Gu Ban during the Eastern Han Dynasty in 105 AD) that “women between the age of 15 and 30 years old, if not married, will have to pay five times the tax”^65^. In addition, as detailed in the Chapter of *Wu Di Ji* (meaning “biography of Emperor Wu”) in *Jin Shu* (meaning “the Book of Jin”; compiled by Xuanling Fang, Suiliang Chu, etc. during the Tang Dynasty from 646 to 648 ad), the emperor of Yan Sima decreed in the winter of 273 ad that “women that are not married off by their parents by the age of 17 years old will be handed over to the local officials to be married off”^66^. During the later dynasties, such as the Western Wei (535 to 556 ad) and the Northern Qi (550 to 577 ad), the laws and regulations became even more strict on marriage age as a result of the huge amount of population loss that was caused by frequent wars^67,68^. In this context, people generally were married at an early age (between 9 to 13 years old) during the Han to Jin Dynasties. For instance, Emperor Hui`s wife, Queen Zhang, was documented to have married him at the age of 12 years old, whereas Emperor Zhao`s wife, Queen Shangguan, married him at the age of ~ 8 years old^65,69^.

According to Chinese historical literature, the peoples and kingdoms of ancient Xinjiang were also in frequent military conflicts and wars during the Han to Jin Dynasties, and some of these wars were for the control of the carven routes of the Silk Road^65,69^. In particular, the studied site of Yingpan was suggested to be an important military station on the Silk Road as military remains such as a beacon tower and fort were both discovered^46,48,70^. Thus, it is very probable that the ancient inhabitants of Yingpan were also married at an early age. Possible supporting evidence is inferred from physical anthropological research which shows that the average age of death at Yingpan was ~ 38.5 years old (n = 38)^71^. This implies that the people of Yingpan would have to get married earlier and give birth to children earlier in order to have enough time to raise the children to adulthood. In conclusion, some individuals of Yingpan had dramatic dietary changes around the age of ~ 9 to ~ 14 years old, and this was possibly related to changes of lifeways and food sources that were caused by the change of an individual`s social role. However, these findings are only preliminary and a larger more detailed study is planned for the future.

## Conclusion

Understanding past breastfeeding, weaning and childhood dietary practices can provide key information about aspects of human life histories and dietary patterns along the Silk Road. Thus, here, using the technique of stable isotope ratio analysis of incremental dentine, this research investigated the early life histories of humans buried in the Yingpan cemetery site in consideration of their social age categories. The Yingpan site was an important Han to Jin Dynasty city that used to be a strategic trading and military location on the Silk Road. The results of this research indicate that there were significant inter-individual differences in terms of the timing and duration of breastfeeding and weaning practices as well as what kind of weaning supplementary foods were consumed in Yingpan. To be specific, among the studied individuals of Yingpan, three (YP4, YP33 and P8) were exclusively breastfed after birth, while, three (YP45, YP35, Yingpan Man) were not. In addition, the post-weaning diets of most Yingpan individuals were relatively stable, but two (YP35 & Yingpan Man) consumed significant amounts of millets during late and post-weaning periods, and displayed frequent dietary changes. In combination with the other findings of millet consumption during infancy, the evidence presented here suggests that the tradition of using millets to feed infants was likely transported to the other regions of the Eurasian continent alongside the Old-World globalization process. Further, seven out of the nine individuals displayed dramatic dietary switches at around 9 to 14 years old, and this may have been related to changes in their social roles during late childhood and/or puberty and marriage.

## Materials and methods

Well-preserved teeth specimens that were not heavily worn: upper canines (UC; n = 2); lower canine (LC; n = 1); lower first pre-molar (LP1; n = 1); lower first molars (LM1; n = 2); lower second molars (LM2; n = 2) were collected from 8 individuals (2 male, 2 female, 4 unknown sex) to investigate breastfeeding, weaning and childhood dietary practices of this site. Sequential samples of incremental dentinal collagen were extracted and collected from the teeth of these individuals following the protocols mentioned in Beaumont et al.^31^ with the modification of demineralizing the teeth at room temperature.

The estimated age of each serial dentinal section was assigned according to the age upon which the crown and the root initiates and completes formation^30^. For example, assuming the crown of the lower first permanent molar (LM1) starts initiating at around 0.3 years old and ceases at ~ 3.5 years old, as referred to in AlQahtani et al.^72,73^, and that six 1 mm dentinal sections are sliced and collected from this area, each sample should then cover approximately a sixth of that age range or timespan, which is ~ 0.53 years old. In this way, the first 1 mm section closest to the crown of this tooth should represent an approximate age range of ~ 0.3 to ~ 0.8 years old and an approximate median age for this dentinal section would be ~ 0.6 years old. According to this, the teeth samples studied here represent a broad age range from birth to childhood and adolescent. To be specific, the upper canine crown initiates formation at ~ 0.6 years old, completes at ~ 5.5 years old, and the apex completes formation at ~ 14.5 years old. The lower canine crown starts formation at ~ 0.9 years old, completes formation at ~ 5.5 years old, and the apex completes formation at ~ 13 years old. The lower first pre-molar crown initiates at ~ 2.5 years old, completes at ~ 6.5 years old and the apex completes formation at ~ 13.5 years old. The lower first molar crown initiates at ~ 0.3 years old, completes at ~ 3.5 years old and the apex completes at ~ 10 years old. The lower second molar crown initiates at ~ 2.5 years old, completes at ~ 8.5 years old, and the apex completes formation at ~ 15.5 years old^30^. The details of this information are listed in Table S2.

However, a note of caution is warranted, as the sampling and aging methods employed here have inevitable limitations and drawbacks. These include: (1) Teeth grow along two axes (the outward apposition of the enamel/dentine matrix away from the dentin-enamel junction, and the downward extension of the matrix towards the root)^74^, but the current method assumes that teeth grow linearly, and the serial dentinal sections are sliced and sampled following a horizontal direction^30^; (2) The rate of apposition and extension change through the development of a tooth, so the 1 mm dentinal sections closer to the crown average a different number of days/weeks/months than the sections closer to the root, but the current method for estimating the age of the serial sections assumes that the dentinal sections from the crown and/or the root grow at even rates; (3) The aging method proposed by Beaumont et al.^30^ is based on the timing of tooth formation described in the Queen Mary University in London (QMUL) London atlas^72,73^, but this database is limited in terms of region, race and era. In this context, the employed method used for sampling and aging human serial dentine will lead to mixtures of isotopic signals with the nearby dentinal sections. The stable isotope values of 1 mm dentinal sections provide isotopic values of dietary input averaged over a period of ~ 3.6 to ~ 10.3 months, which change from the crown to the root. Thus, the estimated age ranges and the estimated median age ranges of the dentinal sections must be considered as approximate and the approximate median ages are used here for convenience.

The extracted dentinal collagen samples were measured in the Archaeological Stable Isotope Laboratory (ASIL), the Department of Archaeology and Anthropology at the University of the Chinese Academy of Sciences, with ~ 0.5 mg of collagen placed into tin capsules for the measurement of δ^13^C and δ^15^N values. The mass spectrometer was an IsoPrime 100 IRMS coupled with the Vario PYRO cube. The stable isotope results were analyzed as the ratio of the heavier isotope to the lighter isotope (^13^C/^12^C or ^15^N/^14^N) and reported as “δ” in parts “per mil (‰) relative to internationally defined standards for carbon (Vienna Pee Dee Belemnite, VPDB) and nitrogen (Ambient Inhalable Reservoir, AIR)^75^. In addition, sulfanilamide was used as a reference material for elemental analysis, IAEA-600 (caffeine), IEAE-N-1 (ammonium sulfate), IAEA-N-2 (ammonium sulfate), IAEA-CH-6 (sucrose), USGS-40 (L-glutamic acid), USGS-41 (L-glutamic acid), USGS-42 (Tibetan human hair powder) and USGS-43 (Indian human hair powder) were used as standards for stable isotope ratio analysis. Among which, USGS-40 (δ^13^C: – 26.39‰; δ^15^N: – 4.52‰) and USGS-41 (δ^13^C: + 37.63‰; δ^15^N: + 47.57‰) were used as standards for two-point calibration of δ^13^C and δ^15^N values. Moreover, for every 10 samples, a collagen lab standard (δ^13^C value of 14.7 ± 0.2‰ and δ^15^N value of 6.9 ± 0.2‰) was also inserted in the run for data monitoring. Approximately 10% of the dentinal samples (n = 22) are measured in duplicates. Following Szpak et al.^76^, analytical precision (uR(w)) was determined to be ± 0.13‰ and ± 0.24‰ respectively for δ^13^C and δ^15^N on the basis of repeated measurements of calibration standards, check standards and sample replicates. Accuracy (u(bias)) was determined to be ± 0.15‰ and ± 0.23‰ respectively for δ^13^C and δ^15^N on the basis of the difference between the observed and known δ values of the check standards and the long-term standard deviations of these check standards. The total analytical uncertainty (Uc) was estimated to be ± 0.20‰ for δ^13^C and ± 0.33‰ for δ^15^N. The measured isotopic results are all listed in Table S2 and are examined throughout the Results and Discussion with descriptive comparisons of the means, SDs and data ranges. In addition, statistical analysis of significant difference is also applied to analyze the data, but the results are only used as complementary evidence to show if there were significant dietary changes through the early life times of the Yingpan humans.

### Ethics declarations

Permission was obtained from appropriate authorities from where the samples were collected and from where the study was carried out. The involved authorities are detailed in the affiliation list of this manuscript with corresponding co-authors representing each affiliation. In addition, this research uses archaeological human samples. Informed consent of using, analysing and publishing these samples (as well as their data) was obtained from the Institute of Archaeology and Cultural Relics of Xinjiang (where the samples are collected). All activities involved in this research are well-complied with the ethical principles, applicable international and national laws as well as the relevant guidelines and regulations.

## Supplementary Information

Supplementary Tables.

## Data Availability

All data needed to evaluate the conclusions in the paper are present in the paper and/or the Supplementary Materials. Additional data related to this paper may be requested from the authors.
