# Supplementary material for: Infancy, childhood, and puberty on the Silk Road revealed with isotopic analysis of incremental dentine
Source: Sci Rep. 2022 Nov 14;12:19494. doi: 10.1038/s41598-022-24119-3 (PMC9663559; doi:10.1038/s41598-022-24119-3)
Supplement: Supplementary file 1 — Supplementary Tables. [file 41598_2022_24119_MOESM1_ESM.doc]

**Supplementary Table** for *Infancy, childhood, and puberty on the Silk Road revealed with isotopic analysis of incremental dentine* by Tingting Wang, Dong Wei, Bing Yi, Hongen Jiang, Wenying Li, Yaowu Hu and Benjamin T. Fuller

**Table S**1. Male and female life courses inferred from Chinese historical literature.

| **Social Age Period** | **Chinese** | **Approximate Age (yrs.)** | **Description** | **Historical Literature*** |
| --- | --- | --- | --- | --- |
| Infancy | Qiang Bao | **0 – ~1** | Baby in swaddling clothes | *Lie Zi* (450 – 375 BC) [^1^](#_ENREF_1) |
| Early Childhood | Hai Ti | **~2 – ~3** | Child that needs to be carried | *Meng Zi (Mencius,* 372 – 289 BC) [^2^](#_ENREF_2) |
| Middle Childhood | Chui Tiao | **Females: ~3 – ~8** | Hair down | *San Guo Zhi* (“Records of the Three Kingdoms”; 280 **–** 290 AD) [^3^](#_ENREF_3) |
|  |  | **Males: ~3 – ~9** |  |  |
| Late Childhood | Zong Jiao | **Females: ~8 – ~14** | Hair in two knots | *Shi Jing* (“The Book of Poetry”; 11^th^ to 6^th^ Century BC) [^4^](#_ENREF_4) |
|  |  | **Males: ~9 – ~14** |  |  |
| Adulthood | Ji Ji | **~15** | Hair tied up with hairpin (bun into womanhood, reach marriageable age or already engaged) | *Li Ji* (“The Book of Rites”; ~80 AD) [^5^](#_ENREF_5) |
|  | Jia Guan;  Ruo Guan | **~20** | Hair tied up with tuinga (bun into adulthood) | *Li Ji* (“The Book of Rites”; ~80AD) [^5^](#_ENREF_5);  *Yi Li* (“Rites”; 770 – 221 BC) [^6^](#_ENREF_6) |

***** = The information presented in this column includes the name of the historical literature, the English meaning of the name, the age upon which the historical literature was complied.

**Table S2. Stable carbon and nitrogen isotope result on serial dentine of Yingpan humans.**

| **Sample No.** | **Context** | **Sex** | **Element** | **Lab No.** | **Distance***  **(mm)** | **Root/ Crown** | **Estimated Age Range (yr) ^#^** | **Estimated Median**  **Age (yr) ^##^** | **δ^13^C**  **(‰)** | **δ^15^N**  **(‰)** | **C%** | **N%** | **Atomic**  **C:N** |
| --- | --- | --- | --- | --- | --- | --- | --- | --- | --- | --- | --- | --- | --- |
| **YP4** | **99BYYM4** | - | Lower  right  first  molar  (LRM1) | AIL1290 | 1 | Crown | 0.3 – 0.8 | 0.6 | –16.9 | 19.7 | 39.7 | 14.5 | 3.2 |
|  |  |  |  | AIL1291 | 2 | Crown | 0.8 – 1.4 | 1.1 | –17.1 | 18.6 | 41.5 | 15.2 | 3.2 |
|  |  |  |  | AIL1292 | 3 | Crown | 1.4 – 1.9 | 1.6 | –17.8 | 16.4 | 42.4 | 15.5 | 3.2 |
|  |  |  |  | AIL1293 | 4 | Crown | 1.9 – 2.4 | 2.2 | –18.1 | 15.7 | 41.8 | 15.4 | 3.2 |
|  |  |  |  | AIL1294 | 5 | Crown | 2.4 – 3.0 | 2.7 | –18.0 | 16.0 | 41.0 | 15.3 | 3.1 |
|  |  |  |  | AIL1295 | 6 | Crown | 3.0 – 3.5 | 3.2 | –17.9 | 16.1 | 40.1 | 14.9 | 3.2 |
|  |  |  |  | AIL1296 | 7 | Root | 3.5 – 4.2 | 3.9 | –17.9 | 16.4 | 41.9 | 15.2 | 3.2 |
|  |  |  |  | AIL1297 | 8 | Root | 4.2 – 4.9 | 4.6 | –17.8 | 16.6 | 41.8 | 15.4 | 3.2 |
|  |  |  |  | AIL1298 | 9 | Root | 4.9 – 5.7 | 5.3 | –18.1 | 17.0 | 41.4 | 15.0 | 3.2 |
|  |  |  |  | AIL1299 | 10 | Root | 5.7 – 6.4 | 6.0 | –18.0 | 16.9 | 40.9 | 15.0 | 3.2 |
|  |  |  |  | AIL1300 | 11 | Root | 6.4 – 7.1 | 6.8 | –18.0 | 17.1 | 42.0 | 15.5 | 3.2 |
|  |  |  |  | AIL1301 | 12 | Root | 7.1 – 7.8 | 7.5 | –18.0 | 17.1 | 40.2 | 14.8 | 3.2 |
|  |  |  |  | AIL1302 | 13 | Root | 7.8 – 8.6 | 8.2 | –18.0 | 17.5 | 40.9 | 15.1 | 3.2 |
|  |  |  |  | AIL1303 | 14 | Root | 8.6 – 9.3 | 8.9 | –18.2 | 17.4 | 40.5 | 15.0 | 3.1 |
|  |  |  |  | AIL1304 | 15 | Root | 9.3 – 10.0 | 9.6 | –18.0 | 17.4 | 40.6 | 14.9 | 3.2 |
| **YP8** | **99BYYM7:L** | Male | Upper  right  canine  (URC) | AIL1305 | 1 | Crown | 0.9 – 1.5 | 0.9 | –15.3 | 17.7 | 38.8 | 14.6 | 3.1 |
|  |  |  |  | AIL1306 | 2 | Crown | 1.5 – 2.1 | 1.5 | –14.6 | 17.8 | 41.1 | 15.2 | 3.2 |
|  |  |  |  | AIL1307 | 3 | Crown | 2.1 – 2.6 | 2.1 | –15.0 | 16.9 | 41.9 | 15.2 | 3.2 |
|  |  |  |  | AIL1308 | 4 | Crown | 2.6 – 3.2 | 2.7 | –15.4 | 16.0 | 42.0 | 15.4 | 3.2 |
|  |  |  |  | AIL1309 | 5 | Crown | 3.2 – 3.8 | 3.4 | –15.7 | 15.6 | 41.9 | 15.3 | 3.2 |
|  |  |  |  | AIL1310 | 6 | Crown | 3.8 – 4.4 | 4.0 | –16.1 | 15.2 | 39.9 | 14.6 | 3.2 |
|  |  |  |  | AIL1311 | 7 | Crown | 4.4 – 4.9 | 4.6 | –16.6 | 15.4 | 41.9 | 15.5 | 3.2 |
|  |  |  |  | AIL1312 | 8 | Crown | 4.9 – 5.5 | 5.2 | –16.9 | 15.3 | 41.8 | 15.3 | 3.2 |
|  |  |  |  | AIL1313 | 9 | Root | 5.5 – 6.1 | 5.8 | –16.7 | 14.9 | 42.1 | 15.2 | 3.2 |
|  |  |  |  | AIL1314 | 10 | Root | 6.1 – 6.6 | 6.3 | –16.4 | 14.9 | 41.3 | 15.0 | 3.2 |
|  |  |  |  | AIL1315 | 11 | Root | 6.6 – 7.2 | 6.9 | –16.2 | 14.8 | 41.2 | 14.7 | 3.3 |
|  |  |  |  | AIL1316 | 12 | Root | 7.2 – 7.8 | 7.5 | –16.0 | 14.6 | 41.3 | 14.9 | 3.2 |
|  |  |  |  | AIL1317 | 13 | Root | 7.8 – 8.3 | 8.0 | –16.1 | 14.7 | 42.2 | 15.0 | 3.3 |
|  |  |  |  | AIL1318 | 14 | Root | 8.3 – 8.9 | 8.6 | –15.9 | 14.7 | 40.0 | 14.6 | 3.2 |
|  |  |  |  | AIL1319 | 15 | Root | 8.9 – 9.4 | 9.2 | –15.8 | 14.5 | 41.5 | 15.0 | 3.2 |
|  |  |  |  | AIL1320 | 16 | Root | 9.4 – 10.0 | 9.7 | –16.0 | 14.3 | 41.0 | 15.0 | 3.2 |
|  |  |  |  | AIL1321 | 17 | Root | 10.0 – 10.6 | 10.3 | –16.0 | 14.7 | 41.5 | 15.1 | 3.2 |
|  |  |  |  | AIL1322 | 18 | Root | 10.6 – 11.1 | 10.8 | –15.8 | 14.9 | 41.7 | 15.3 | 3.2 |
|  |  |  |  | AIL1323 | 19 | Root | 11.1 – 11.7 | 11.4 | –15.7 | 14.6 | 41.7 | 15.1 | 3.2 |
|  |  |  |  | AIL1324 | 20 | Root | 11.7 – 12.3 | 12.0 | –16.1 | 15.0 | 41.1 | 15.1 | 3.2 |
|  |  |  |  | AIL1325 | 21 | Root | 12.3 – 12.8 | 12.5 | –16.1 | 15.3 | 41.5 | 15.1 | 3.2 |
|  |  |  |  | AIL1326 | 22 | Root | 12.8 – 13.4 | 13.1 | –16.0 | 15.1 | 41.3 | 15.2 | 3.2 |
|  |  |  |  | AIL1327 | 23 | Root | 13.4 – 13.9 | 13.7 | –16.4 | 15.8 | 61.7 | 22.7 | 3.2 |
|  |  |  |  | AIL1328 | 24 | Root | 13.9 – 14.5 | 14.2 | –16.5 | 15.9 | 83.1 | 30.0 | 3.2 |
| **YP13** | **99BYYM9:2** | - | Lower  left  second  molar  (LLM2) | AIL1111 | 1 | Crown | 2.5 – 3.3 | 2.9 | –15.0 | 14.2 | 42.0 | 15.5 | 3.2 |
|  |  |  |  | AIL1112 | 2 | Crown | 3.3 – 4.0 | 3.6 | –15.8 | 13.5 | 42.2 | 15.4 | 3.2 |
|  |  |  |  | AIL1113 | 3 | Crown | 4.0 – 4.8 | 4.4 | –16.3 | 13.7 | 42.3 | 15.5 | 3.2 |
|  |  |  |  | AIL1114 | 4 | Crown | 4.8 – 5.5 | 5.1 | –16.3 | 13.7 | 34.5 | 12.7 | 3.2 |
|  |  |  |  | AIL1115 | 5 | Crown | 5.5 – 6.3 | 5.9 | –16.2 | 13.6 | 35.6 | 13.1 | 3.2 |
|  |  |  |  | AIL1116 | 6 | Crown | 6.3 – 7.0 | 6.6 | –15.7 | 13.4 | 41.6 | 15.2 | 3.2 |
|  |  |  |  | AIL1117 | 7 | Crown | 7.0 – 7.8 | 7.4 | –15.4 | 13.5 | 42.4 | 15.5 | 3.2 |
|  |  |  |  | AIL1118 | 8 | Crown | 7.8 – 8.5 | 8.1 | –15.2 | 13.4 | 42.2 | 15.4 | 3.2 |
|  |  |  |  | AIL1119 | 9 | Root | 8.5 – 9.1 | 8.8 | –15.3 | 13.6 | 41.8 | 15.1 | 3.2 |
|  |  |  |  | AIL1120 | 10 | Root | 9.1 – 9.7 | 9.4 | –15.4 | 13.4 | 42.1 | 15.5 | 3.2 |
|  |  |  |  | AIL1121 | 11 | Root | 9.7 – 10.3 | 10.0 | –15.2 | 13.7 | 39.5 | 14.4 | 3.2 |
|  |  |  |  | AIL1122 | 12 | Root | 10.3 – 10.8 | 10.5 | –15.3 | 13.6 | 36.8 | 13.5 | 3.2 |
|  |  |  |  | AIL1123 | 13 | Root | 10.8 – 11.4 | 11.1 | –15.3 | 14.1 | 41.1 | 15.1 | 3.2 |
|  |  |  |  | AIL1124 | 14 | Root | 11.4 – 12.0 | 11.7 | –15.1 | 13.9 | 41.2 | 15.1 | 3.2 |
|  |  |  |  | AIL1125 | 15 | Root | 12.0 – 12.6 | 12.3 | –15.3 | 13.9 | 41.9 | 15.3 | 3.2 |
|  |  |  |  | AIL1126 | 16 | Root | 12.6 – 13.2 | 12.9 | –14.8 | 14.4 | 41.6 | 15.2 | 3.2 |
|  |  |  |  | AIL1127 | 17 | Root | 13.2 – 13.8 | 13.5 | –14.8 | 14.5 | 41.0 | 15.1 | 3.2 |
|  |  |  |  | AIL1128 | 18 | Root | 13.8 – 14.3 | 14.0 | –14.6 | 14.6 | 41.9 | 15.4 | 3.2 |
|  |  |  |  | AIL1129 | 19 | Root | 14.3 – 14.9 | 14.6 | –15.4 | 14.8 | 39.8 | 15.0 | 3.1 |
|  |  |  |  | AIL1130 | 20 | Root | 14.9 – 15.5 | 15.2 | –15.5 | 15.8 | 41.4 | 15.1 | 3.2 |
| **YP23** | **99BYYM23** | Male | Lower  left  first  pre–molar  (LLP1) | AIL1329 | 1 | Crown | 2.5 – 3.2 | 2.8 | –17.2 | 15.6 | 37.0 | 13.6 | 3.2 |
|  |  |  |  | AIL1330 | 2 | Crown | 3.2 – 3.8 | 3.4 | –17.3 | 15.2 | 51.6 | 18.8 | 3.2 |
|  |  |  |  | AIL1331 | 3 | Crown | 3.8 – 4.5 | 3.9 | –17.7 | 15.2 | 47.6 | 17.4 | 3.2 |
|  |  |  |  | AIL1332 | 4 | Crown | 4.5 – 5.2 | 4.5 | –17.5 | 15.6 | 27.4 | 10.0 | 3.2 |
|  |  |  |  | AIL1333 | 5 | Crown | 5.2 – 5.8 | 5.1 | –17.2 | 15.3 | 47.6 | 17.4 | 3.2 |
|  |  |  |  | AIL1334 | 6 | Crown | 5.8 – 6.5 | 5.6 | –17.4 | 15.4 | 43.1 | 15.8 | 3.2 |
|  |  |  |  | AIL1335 | 7 | Crown | 6.5 – 6.9 | 6.2 | –17.5 | 15.5 | 44.0 | 16.3 | 3.1 |
|  |  |  |  | AIL1336 | 8 | Root | 6.9 – 7.2 | 6.7 | –17.5 | 14.9 | 39.0 | 14.3 | 3.2 |
|  |  |  |  | AIL1337 | 9 | Root | 7.2 – 7.6 | 7.1 | –17.4 | 14.8 | 41.6 | 15.3 | 3.2 |
|  |  |  |  | AIL1338 | 10 | Root | 7.6 – 8.0 | 7.5 | –17.3 | 15.1 | 32.4 | 11.9 | 3.2 |
|  |  |  |  | AIL1339 | 11 | Root | 8.0 – 8.3 | 7.9 | –17.1 | 15.9 | 52.5 | 19.0 | 3.2 |
|  |  |  |  | AIL1340 | 12 | Root | 8.3 – 8.7 | 8.3 | –16.8 | 15.3 | 34.8 | 12.9 | 3.1 |
|  |  |  |  | AIL1341 | 13 | Root | 8.7 – 9.1 | 8.6 | –16.8 | 15.5 | 39.4 | 14.5 | 3.2 |
|  |  |  |  | AIL1342 | 14 | Root | 9.1 – 9.4 | 9.0 | –16.8 | 15.5 | 40.6 | 14.9 | 3.2 |
|  |  |  |  | AIL1343 | 15 | Root | 9.4 – 9.8 | 9.4 | –16.8 | 15.3 | 40.4 | 14.7 | 3.2 |
|  |  |  |  | AIL1344 | 16 | Root | 9.8 – 10.2 | 9.8 | –16.8 | 15.8 | 40.9 | 15.0 | 3.2 |
|  |  |  |  | AIL1345 | 17 | Root | 10.2 – 10.6 | 10.2 | –17.0 | 15.3 | 41.1 | 15.1 | 3.2 |
|  |  |  |  | AIL1346 | 18 | Root | 10.6 – 10.9 | 10.6 | –17.2 | 16.2 | 41.0 | 15.1 | 3.2 |
|  |  |  |  | AIL1347 | 19 | Root | 10.9 – 11.3 | 11.0 | –17.3 | 15.7 | 40.3 | 14.9 | 3.2 |
|  |  |  |  | AIL1348 | 20 | Root | 11.3 – 11.7 | 11.4 | –17.5 | 16.1 | 40.1 | 14.6 | 3.2 |
|  |  |  |  | AIL1349 | 21 | Root | 11.7 – 12.0 | 11.8 | –17.5 | 15.7 | 40.9 | 15.0 | 3.2 |
|  |  |  |  | AIL1350 | 22 | Root | 12.0 – 12.4 | 12.1 | –17.4 | 16.3 | 41.1 | 14.8 | 3.2 |
|  |  |  |  | AIL1351 | 23 | Root | 12.4 – 12.8 | 12.5 | –17.7 | 16.2 | 40.3 | 15.0 | 3.1 |
|  |  |  |  | AIL1352 | 24 | Root | 12.8 – 13.1 | 12.9 | –17.7 | 16.4 | 40.5 | 14.9 | 3.2 |
|  |  |  |  | AIL1353 | 25 | Root | 13.1 – 13.5 | 13.3 | –17.9 | 15.9 | 29.3 | 11.1 | 3.1 |
| **YP28** | **99BYYM31** | - | Lower  right  second  molar  (LRM2) | AIL1464 | 1 | Crown | 2.5 – 3.4 | 2.9 | –16.1 | 15.8 | 39.7 | 14.7 | 3.1 |
|  |  |  |  | AIL1465 | 2 | Crown | 3.4 – 4.2 | 3.8 | –15.9 | 15.9 | 41.7 | 15.5 | 3.1 |
|  |  |  |  | AIL1466 | 3 | Crown | 4.2 – 5.1 | 4.6 | –16.0 | 16.0 | 41.7 | 15.3 | 3.2 |
|  |  |  |  | AIL1467 | 4 | Crown | 5.1 – 5.9 | 5.5 | –16.1 | 15.8 | 40.8 | 15.1 | 3.2 |
|  |  |  |  | AIL1468 | 5 | Crown | 5.9 – 6.8 | 6.4 | –16.0 | 15.4 | 38.2 | 13.9 | 3.2 |
|  |  |  |  | AIL1469 | 6 | Crown | 6.8 – 7.6 | 7.2 | –15.8 | 14.9 | 34.6 | 12.7 | 3.2 |
|  |  |  |  | AIL1470 | 7 | Crown | 7.6 – 8.5 | 8.1 | –15.4 | 15.3 | 20.7 | 7.5 | 3.2 |
|  |  |  |  | AIL1471 | 8 | Root | 8.5 – 9.1 | 8.8 | –15.1 | 15.3 | 34.4 | 12.5 | 3.2 |
|  |  |  |  | AIL1472 | 9 | Root | 9.1 – 9.7 | 9.4 | –15.3 | 15.1 | 39.6 | 14.3 | 3.2 |
|  |  |  |  | AIL1473 | 10 | Root | 9.7 – 10.3 | 10 | –15.1 | 15.5 | 41.0 | 14.9 | 3.2 |
|  |  |  |  | AIL1474 | 11 | Root | 10.3 – 10.8 | 10.5 | –14.9 | 15.7 | 41.4 | 15.1 | 3.2 |
|  |  |  |  | AIL1475 | 12 | Root | 10.8 – 11.4 | 11.1 | –15.0 | 16.3 | 42.4 | 15.4 | 3.2 |
|  |  |  |  | AIL1476 | 13 | Root | 11.4 – 12.0 | 11.7 | –15.6 | 15.7 | 38.0 | 13.8 | 3.2 |
|  |  |  |  | AIL1477 | 14 | Root | 12.0 – 12.6 | 12.3 | –16.5 | 16.2 | 38.8 | 13.7 | 3.3 |
|  |  |  |  | AIL1478 | 15 | Root | 12.6 – 13.2 | 12.9 | –16.0 | 16.6 | 41.3 | 15.2 | 3.2 |
|  |  |  |  | AIL1479 | 16 | Root | 13.2 – 13.8 | 13.5 | –16.3 | 16.1 | 40.8 | 15.1 | 3.2 |
|  |  |  |  | AIL1480 | 17 | Root | 13.8 – 14.3 | 14 | –16.1 | 15.7 | 41.5 | 15.4 | 3.1 |
|  |  |  |  | AIL1481 | 18 | Root | 14.3 – 14.9 | 14.6 | –16.2 | 15.5 | 41.9 | 15.4 | 3.2 |
|  |  |  |  | AIL1482 | 19 | Root | 14.9 – 15.5 | 15.2 | –16.1 | 16.8 | 41.2 | 14.7 | 3.3 |
| **YP33** | **99BYYM42** | Female | Lower  right  first  molar  (LRM1) | AIL1354 | 1 | Crown | 0.3 – 0.8 | 0.6 | –14.6 | 19.9 | 39.5 | 14.6 | 3.2 |
|  |  |  |  | AIL1355 | 2 | Crown | 0.8 – 1.4 | 1.1 | –14.9 | 19.1 | 40.8 | 15.0 | 3.2 |
|  |  |  |  | AIL1356 | 3 | Crown | 1.4 – 1.9 | 1.6 | –15.8 | 17.3 | 41.8 | 15.3 | 3.2 |
|  |  |  |  | AIL1357 | 4 | Crown | 1.9 – 2.4 | 2.2 | –16.2 | 16.4 | 38.9 | 14.3 | 3.2 |
|  |  |  |  | AIL1358 | 5 | Crown | 2.4 – 3.0 | 2.7 | –16.1 | 15.5 | 42.1 | 15.4 | 3.2 |
|  |  |  |  | AIL1359 | 6 | Crown | 3.0 – 3.5 | 3.2 | –15.7 | 15.3 | 42.1 | 15.4 | 3.2 |
|  |  |  |  | AIL1360 | 7 | Root | 3.5 – 4.0 | 3.8 | –15.6 | 15.6 | 41.2 | 15.0 | 3.2 |
|  |  |  |  | AIL1361 | 8 | Root | 4.0 – 4.6 | 4.3 | –15.7 | 15.4 | 41.2 | 15.3 | 3.1 |
|  |  |  |  | AIL1362 | 9 | Root | 4.6 – 5.1 | 4.9 | –15.5 | 15.8 | 42.0 | 15.4 | 3.2 |
|  |  |  |  | AIL1363 | 10 | Root | 5.1 – 5.7 | 5.4 | –15.6 | 15.8 | 41.2 | 15.2 | 3.2 |
|  |  |  |  | AIL1364 | 11 | Root | 5.7 – 6.2 | 5.9 | –15.3 | 15.8 | 41.1 | 15.1 | 3.2 |
|  |  |  |  | AIL1365 | 12 | Root | 6.2 – 6.8 | 6.5 | –15.4 | 16.1 | 40.0 | 14.9 | 3.1 |
|  |  |  |  | AIL1366 | 13 | Root | 6.8 – 7.3 | 7.0 | –15.7 | 16.4 | 41.1 | 15.2 | 3.2 |
|  |  |  |  | AIL1367 | 14 | Root | 7.3 – 7.8 | 7.6 | –15.8 | 16.2 | 41.3 | 15.1 | 3.2 |
|  |  |  |  | AIL1368 | 15 | Root | 7.8 – 8.4 | 8.1 | –16.7 | 16.5 | 41.1 | 15.2 | 3.2 |
|  |  |  |  | AIL1369 | 16 | Root | 8.4 – 8.9 | 8.6 | –17.0 | 16.4 | 40.4 | 14.8 | 3.2 |
|  |  |  |  | AIL1370 | 17 | Root | 8.9 – 9.5 | 9.2 | –16.6 | 16.6 | 39.4 | 14.5 | 3.2 |
|  |  |  |  | AIL1371 | 18 | Root | 9.5 – 10.0 | 9.7 | –16.4 | 16.8 | 40.8 | 15.0 | 3.2 |
| **YP35** | **99BYYM46** | - | Upper  left  canine  (ULC) | AIL1372 | 1 | Crown | 0.6 – 1.3 | 1.0 | –16.8 | 18.8 | 40.9 | 15.2 | 3.1 |
|  |  |  |  | AIL1373 | 2 | Crown | 1.3 – 2.0 | 1.7 | –16.5 | 19.1 | 40.6 | 14.9 | 3.2 |
|  |  |  |  | AIL1374 | 3 | Crown | 2.0 – 2.7 | 2.4 | –16.6 | 17.9 | 40.0 | 14.8 | 3.2 |
|  |  |  |  | AIL1375 | 4 | Crown | 2.7 – 3.4 | 3.1 | –16.4 | 16.9 | 41.1 | 15.2 | 3.1 |
|  |  |  |  | AIL1376 | 5 | Crown | 3.4 – 4.1 | 3.8 | –15.6 | 16.9 | 41.2 | 15.2 | 3.2 |
|  |  |  |  | AIL1377 | 6 | Crown | 4.1 – 4.8 | 4.5 | –14.9 | 17.6 | 41.4 | 15.3 | 3.1 |
|  |  |  |  | AIL1378 | 7 | Crown | 4.8 – 5.5 | 5.2 | –15.3 | 17.8 | 40.8 | 14.9 | 3.2 |
|  |  |  |  | AIL1379 | 8 | Root | 5.5 – 6.0 | 5.7 | –15.6 | 17.7 | 41.0 | 14.9 | 3.2 |
|  |  |  |  | AIL1380 | 9 | Root | 6.0 – 6.4 | 6.2 | –15.3 | 17.5 | 42.2 | 15.6 | 3.2 |
|  |  |  |  | AIL1381 | 10 | Root | 6.4 – 6.9 | 6.7 | –15.3 | 18.0 | 41.3 | 15.0 | 3.2 |
|  |  |  |  | AIL1382 | 11 | Root | 6.9 – 7.4 | 7.2 | –15.4 | 17.3 | 40.2 | 14.7 | 3.2 |
|  |  |  |  | AIL1383 | 12 | Root | 7.4 – 7.9 | 7.6 | –15.9 | 17.6 | 41.3 | 15.0 | 3.2 |
|  |  |  |  | AIL1384 | 13 | Root | 7.9 – 8.3 | 8.1 | –16.4 | 17.6 | 39.9 | 14.9 | 3.1 |
|  |  |  |  | AIL1385 | 14 | Root | 8.3 – 8.8 | 8.6 | –16.5 | 17.5 | 40.9 | 14.9 | 3.2 |
|  |  |  |  | AIL1386 | 15 | Root | 8.8 – 9.3 | 9.1 | –16.1 | 17.5 | 40.5 | 14.9 | 3.2 |
|  |  |  |  | AIL1387 | 16 | Root | 9.3 – 9.8 | 9.5 | –15.1 | 17.8 | 40.3 | 14.8 | 3.2 |
|  |  |  |  | AIL1388 | 17 | Root | 9.8 – 10.2 | 10.0 | –15.0 | 17.4 | 40.3 | 14.7 | 3.2 |
|  |  |  |  | AIL1389 | 18 | Root | 10.2 – 10.7 | 10.5 | –14.7 | 17.7 | 40.8 | 15.0 | 3.2 |
|  |  |  |  | AIL1390 | 19 | Root | 10.7 – 11.2 | 10.9 | –15.0 | 17.3 | 40.6 | 15.0 | 3.2 |
|  |  |  |  | AIL1391 | 20 | Root | 11.2 – 11.7 | 11.4 | –15.9 | 16.1 | 41.0 | 15.0 | 3.2 |
|  |  |  |  | AIL1392 | 21 | Root | 11.7 – 12.1 | 11.9 | –16.4 | 15.3 | 41.6 | 15.1 | 3.2 |
|  |  |  |  | AIL1393 | 22 | Root | 12.1 – 12.6 | 12.4 | –16.0 | 15.5 | 41.3 | 15.1 | 3.2 |
|  |  |  |  | AIL1394 | 23 | Root | 12.6 – 13.1 | 12.8 | –15.9 | 16.8 | 40.6 | 15.0 | 3.2 |
|  |  |  |  | AIL1395 | 24 | Root | 13.1 – 13.6 | 13.3 | –15.1 | 17.5 | 41.3 | 15.1 | 3.2 |
|  |  |  |  | AIL1396 | 25 | Root | 13.6 – 14.0 | 13.8 | –15.8 | 18.1 | 41.1 | 15.2 | 3.2 |
|  |  |  |  | AIL1397 | 26 | Root | 14.0 – 14.5 | 14.3 | –15.9 | 17.8 | 40.2 | 14.7 | 3.2 |
| **YP45** | **95BYYM14** | Female | Lower  left  canine  (LLC) | AIL1178 | 1 | Crown | 0.9 – 1.6 | 1.2 | –17.1 | 18.5 | 40.3 | 15.3 | 3.1 |
|  |  |  |  | AIL1179 | 2 | Crown | 1.6 – 2.2 | 1.9 | –16.6 | 18.6 | 40.4 | 15.2 | 3.1 |
|  |  |  |  | AIL1180 | 3 | Crown | 2.2 – 2.9 | 2.5 | –16.3 | 18.1 | 41.4 | 15.2 | 3.2 |
|  |  |  |  | AIL1181 | 4 | Crown | 2.9 – 3.5 | 3.2 | –16.8 | 16.4 | 41.6 | 15.3 | 3.2 |
|  |  |  |  | AIL1182 | 5 | Crown | 3.5 – 4.2 | 3.9 | –17.2 | 15.6 | 41.5 | 15.3 | 3.2 |
|  |  |  |  | AIL1183 | 6 | Crown | 4.2 – 4.8 | 4.5 | –16.8 | 16.1 | 41.2 | 15.2 | 3.2 |
|  |  |  |  | AIL1184 | 7 | Crown | 4.8 – 5.5 | 5.2 | –16.6 | 16.5 | 41.8 | 15.3 | 3.2 |
|  |  |  |  | AIL1185 | 8 | Root | 5.5 – 5.9 | 5.7 | –17.0 | 16.2 | 41.1 | 15.2 | 3.2 |
|  |  |  |  | AIL1186 | 9 | Root | 5.9 – 6.3 | 6.1 | –17.2 | 16.1 | 42.2 | 15.5 | 3.2 |
|  |  |  |  | AIL1187 | 10 | Root | 6.3 – 6.8 | 6.5 | –17.2 | 15.3 | 41.5 | 15.3 | 3.2 |
|  |  |  |  | AIL1188 | 11 | Root | 6.8 – 7.2 | 7 | –17.5 | 15.0 | 41.3 | 15.3 | 3.2 |
|  |  |  |  | AIL1189 | 12 | Root | 7.2 – 7.6 | 7.4 | –17.3 | 14.9 | 41.7 | 15.3 | 3.2 |
|  |  |  |  | AIL1190 | 13 | Root | 7.6 – 8.0 | 7.8 | –17.0 | 14.7 | 41.6 | 15.4 | 3.2 |
|  |  |  |  | AIL1191 | 14 | Root | 8.0 – 8.4 | 8.2 | –16.8 | 14.6 | 41.5 | 15.3 | 3.2 |
|  |  |  |  | AIL1192 | 15 | Root | 8.4 – 8.8 | 8.6 | –16.9 | 14.4 | 41.6 | 15.3 | 3.2 |
|  |  |  |  | AIL1193 | 16 | Root | 8.8 – 9.3 | 9 | –17.1 | 14.1 | 40.9 | 15.1 | 3.2 |
|  |  |  |  | AIL1194 | 17 | Root | 9.3 – 9.7 | 9.5 | –16.9 | 14.1 | 27.2 | 10.0 | 3.2 |
|  |  |  |  | AIL1195 | 18 | Root | 9.7 – 10.1 | 9.9 | –16.8 | 14.0 | 30.0 | 11.1 | 3.1 |
|  |  |  |  | AIL1196 | 19 | Root | 10.1 – 10.5 | 10.3 | –16.7 | 13.9 | 44.3 | 16.2 | 3.2 |
|  |  |  |  | AIL1197 | 20 | Root | 10.5 – 10.9 | 10.7 | –16.9 | 14.0 | 27.6 | 10.0 | 3.2 |
|  |  |  |  | AIL1198 | 21 | Root | 10.9 – 11.3 | 11.1 | –16.5 | 13.9 | 33.1 | 12.2 | 3.2 |
|  |  |  |  | AIL1199 | 22 | Root | 11.3 – 11.8 | 11.5 | –16.3 | 14.4 | 34.5 | 12.8 | 3.1 |
|  |  |  |  | AIL1200 | 23 | Root | 11.8 – 12.2 | 12 | –16.6 | 13.8 | 35.0 | 12.9 | 3.2 |
|  |  |  |  | AIL1201 | 24 | Root | 12.2 – 12.6 | 12.4 | –15.8 | 14.5 | 41.0 | 14.9 | 3.2 |
|  |  |  |  | AIL1202 | 25 | Root | 12.6 – 13.0 | 12.8 | –16.8 | 15.1 | 40.8 | 15.1 | 3.2 |
| **YP46^** | **99BYYM15** | Male | Upper  right  first  molar  (URM1) | AIL1398 | 1 | Crown | 0.3 – 0.8 | 0.5 | –16.8 | 19.7 | 40.7 | 14.7 | 3.2 |
|  |  |  |  | AIL1399 | 2 | Crown | 0.8 – 1.2 | 1.0 | –16.9 | 19.1 | 41.9 | 15 | 3.2 |
|  |  |  |  | AIL1400 | 3 | Crown | 1.2 – 1.7 | 1.4 | –17.0 | 18.5 | 35.3 | 12.7 | 3.3 |
|  |  |  |  | AIL1401 | 4 | Crown | 1.7 – 2.1 | 1.9 | –16.9 | 17.8 | 29.6 | 10.7 | 3.2 |
|  |  |  |  | AIL1402 | 5 | Crown | 2.1 – 2.6 | 2.4 | –16.6 | 17.2 | 30.1 | 10.9 | 3.2 |
|  |  |  |  | AIL1403 | 6 | Crown | 2.6 – 3.0 | 2.8 | –15.9 | 17.2 | 32.7 | 11.8 | 3.2 |
|  |  |  |  | AIL1404 | 7 | Crown | 3.0 – 3.5 | 3.3 | –15.7 | 17.1 | 33.5 | 12.1 | 3.2 |
|  |  |  |  | AIL1405 | 8 | Root | 3.5 – 3.9 | 3.7 | –15.5 | 17.3 | 39.4 | 14.1 | 3.3 |
|  |  |  |  | AIL1406 | 9 | Root | 3.9 – 4.4 | 4.1 | –15.3 | 17.5 | 24.4 | 8.8 | 3.2 |
|  |  |  |  | AIL1407 | 10 | Root | 4.4 – 4.8 | 4.6 | –15.7 | 17.2 | 29.6 | 10.8 | 3.2 |
|  |  |  |  | AIL1408 | 11 | Root | 4.8 – 5.2 | 5.0 | –15.8 | 17.6 | 25.2 | 9.2 | 3.2 |
|  |  |  |  | AIL1409 | 12 | Root | 5.2 – 5.6 | 5.4 | –16.1 | 17.9 | 30 | 10.8 | 3.2 |
|  |  |  |  | AIL1410 | 13 | Root | 5.6 – 6.1 | 5.9 | –16.5 | 17.6 | 23.7 | 8.7 | 3.2 |
|  |  |  |  | AIL1411 | 14 | Root | 6.1 – 6.5 | 6.3 | –16.7 | 17.8 | 23.3 | 8.5 | 3.2 |
|  |  |  |  | AIL1412 | 15 | Root | 6.5 – 6.9 | 6.7 | –16.6 | 17.7 | 25.5 | 9 | 3.3 |
|  |  |  |  | AIL1413 | 16 | Root | 6.9 – 7.4 | 7.1 | –16.0 | 17.2 | 24.5 | 8.9 | 3.2 |
|  |  |  |  | AIL1414 | 17 | Root | 7.4 – 7.8 | 7.6 | –15.8 | 17.3 | 36.1 | 13.1 | 3.2 |
|  |  |  |  | AIL1415 | 18 | Root | 7.8 – 8.2 | 8.0 | –15.8 | 17.7 | 41.8 | 15.2 | 3.2 |
|  |  |  |  | AIL1416 | 19 | Root | 8.2 – 8.6 | 8.4 | –15.4 | 17.4 | 37.3 | 13.6 | 3.2 |
|  |  |  |  | AIL1417 | 20 | Root | 8.6 – 9.1 | 8.9 | –15.7 | 17.4 | 39.7 | 14.6 | 3.2 |
|  |  |  |  | AIL1418 | 21 | Root | 9.1 – 9.5 | 9.3 | –15.4 | 17.0 | 39.3 | 14.4 | 3.2 |

Notes: “-” represents data or information that are unavailable;

“Distance*****” represents the distance of the serial section from the crown;

“Age Range (yr)^#^” represents the approximate age range that each serial section formed according to Beaumont and Montgomery [^7^](#_ENREF_7);

“Median Age^##^” represents the approximate median age that each serial section formed according to Beaumont and Montgomery [^7^](#_ENREF_7)；

“YP46**^**” represents that the serial dentine isotopic data of individual YP46 are previously published in Wang et al. [^8^](#_ENREF_8).

REFERENCES

1 [Warring States Period] Lie, Y. Lie Zi. (Zhonghua Book Company, 2018).

2 [Warring States Period] Mencius. Meng Zi. (Zhonghua Book Company, 2017).

3 [Jin Dynasty] Chen, S. Records of the Three Kingdoms (San Guo Zhi). (Zhonghua Book Company, 2011).

4 [Zhou Dynasty] Yin, J. & Confucius. The Book of poetry (Shi Jing). (Zhonghua Book Company, 2015).

5 [Han Dynasty] Dai, S. The Book of Rites (Li Ji). (Zhonghua Book Company, 2017).

6 [Zhou Dynasty] Confucius. Rites (Yi Li). (Zhonghua Book Company, 2012).

7 Beaumont, J. & Montgomery, J. Oral histories: a simple method of assigning chronological age to isotopic values from human dentine collagen. *Annals of Human Biology* **42**, 407-414, doi: 10.3109/03014460.2015.1045027 (2015).

8 Wang, T. *et al.* Revealing lost secrets about Yingpan Man and the Silk Road. *Scientific Reports* **12**, 669, doi:10.1038/s41598-021-04383-5 (2022).
